# Plant agronomy, leaf ecophysiology, yield and quality data of interspecific grafted *Coffea arabica* across an elevation gradient

**DOI:** 10.1016/j.dib.2023.109560

**Published:** 2023-09-12

**Authors:** Athina Koutouleas, Conor Blunt, Aljoša Bregar, Jon Kehlet Hansen, Anders Ræbild, Hervé Etienne, Frédéric Georget

**Affiliations:** a Department of Geosciences and Natural Resource Management, University of Copenhagen, Rolighedsvej 23, 1958 Frederiksberg C, Denmark; b CIRAD (Centre de Coopération Internationale en Recherche Agronomique pour le Développement), UMR DIADE, F-34398 Montpellier, France; c UMR DIADE (Diversity, Adaptation, Development of Plants), University of Montpellier, CIRAD, IRD, F-34398 Montpellier, France

**Keywords:** Agroforestry, Agronomy, Artificial Shade, *Coffea canephora*, Coffee, Costa Rica, Field Data, Nemaya, Photosynthesis and Rootstock

## Abstract

In-field data were collected in Costa Rica between 2018–2021 on newly planted grafted and non-grafted coffee plants grown under artificial shade nets and across an elevation gradient (1050, 1250 and 1450 m.a.s.l). The coffee plants consisted of *Coffea arabica* F1 hybrid plants (‘H3 *i.e.* Caturra cv. X Ethiopian 531’), which were derived from a somatic embryogenesis clonal propagation process, an American *C. arabica* pure line (‘Villa Sarchi’) and *C. canephora* ‘Nemaya’ (the latter two both being produced by seed). Data from eight different coffee types (including these three genotypes) and different grafting combinations (including reverse and auto-grafting) were collected. Data concerned plant traits such as grafting compatibility (plant collar diameters above and below graft union), agronomic characteristics (aerial and root traits), leaf ecophysiology (leaf gas-exchange and chlorophyll fluorescence), yield and quality attributes (bean size, peaberry percentage, WB100 and SCA note). Climate data were also included for comparison on the farm plots along the elevation gradient. Linear mixed models were used to test for effects of elevation (test sites), coffee types (grafted or non-grafted combinations) and interaction between coffee types and elevations. Least square mean estimates were calculated for significant fixed effects and Tukey tests applied for pairwise tests. A tangential hyperbola curve was used to analyse leaf gas-exchange data. These datasets and R scripts can be re-used as a guide for future analyses concerning coffee agronomy or eco-physiological interactions for other plant species. Other potential re-uses could be meta-analyses aimed at comparing coffee yield, quality, or other agronomic traits across different environmental conditions (such as under shade of an agroforestry system or across different elevation sites).

Specifications Table

**Table:** 

Subject	Agricultural Sciences (Horticulture)
Specific subject area	Interspecific grafting of coffee plants across an elevation gradient and under shading mimicking an agroforestry system.
Type of data	Table Graph Figure
How the data were acquired	**Agronomic data:** manual collection in field. **Coffee yield:** weight of successive harvest of green beans. **Gas-exchange**: CIRAS-1 and 3 (PP-systems, Hitchin, UK and PP-systems, Amesbury, MA, US). **Chlorophyll fluorescence:** Pocket-PEA fluorimeter (Hansatech Instruments Ltd., UK). **Bean traits:** sieved with mesh size 15/64 to 17/64 inches and physically assessed. **Cup quality:** Cup testing following the Specialty Coffee Association (SCA) protocol.
Data format	Raw R scripts
Description of data collection	All data were collected from a commercial coffee farm in Costa Rica covering three different elevations (1050, 1250 and 1450 m.a.s.l.) by in-field researchers. Fourteen different plant replicates were grown for each coffee-grafted combination. Data from plant replicates which died during any part of the experimental period (2018–2021) were excluded from the dataset.
Data source location	San Pedro de Poas Alajuela Costa Rica Plot 1 (1050 m.a.s.l): 10°5′2.07′'N 84°13′50.75′'O Plot 2 (1250 m.a.s.l): 10°5′35.79′'N 84°14′1.86′'O Plot 3 (1500 m.a.s.l): 10°6′35.14′'N 84°13′32.44′'O
Data accessibility	**Repository name:** Open Science Framework **Data identification number:** g23jf **Direct URL to data:** https://osf.io/g23jf/?view_only=af122c701e7144b8af88bdc82aea9df2. **Instructions for accessing these data:** use URL and download files
Related research article	[1] Koutouleas A, Blunt C, Bregar A, Hansen JH, Ræbild A, Etienne H, Georget F. Effects of interspecific grafting of *Coffea arabica* and elevation on coffee growth, yield, and quality attributes in Costa Rica. Sci. Hortic. 2023 Oct:320. https://doi.org/10.1016/j.scienta.2023.112162.

## 1 Value of the Data

• These data can be used in future studies or farmer recommendations concerning coffee grafting; the varieties/hybrids: ‘Villa Sarchi’, ‘H3 (Caturra × Ethiopian 531)’, ‘Nemaya’; shade or elevation effects in coffee farming.
• The individual datasets provided are particularly useful to researchers, agronomists and farmer extension services who would like to assess either the physiology, agronomy, yield and/or quality of different coffee varieties/hybrids when grafted with a well-known nematode-resistant rootstock.
• These data can be utilized in meta-analyses (such as in [2]), surveys or technical reports. Researchers may also use these data to help guide future coffee field trials which involve the same coffee varieties/hybrids or aimed at a better understanding of the effects of interspecific grafting or of the effects of elevation on grafting success

## 2 Objective

The context behind the generation of this dataset was to examine the effects of interspecific grafting of two *Coffea arabica* genotypes with nematode-resistant rootstocks from the *C. canephora* species (“Nemaya cv.”). These grafted coffee types were tested under a shaded environment as well as along an altitudinal gradient used to create a proxy for temperature. This field design was intended to mimic an agroforestry coffee farming system which has recently received a great deal of attention as being a “nature-based” approach against climate change [3]. The central hypothesis that interspecific grafting of *C. arabica* onto *C. canephora* rootstock would impact coffee growth, yield, and quality attributes to varying degrees across an elevation gradient; the lower altitude site being more suitable for the grafted coffee types with *C. canephora* as rootstocks, compared to the higher altitude sites. This data article provides complete raw data and R-scripts used for analysis of the original research article.

## 3 Data Description

### 3.1 Overview of data sources

**Fig. 1:**
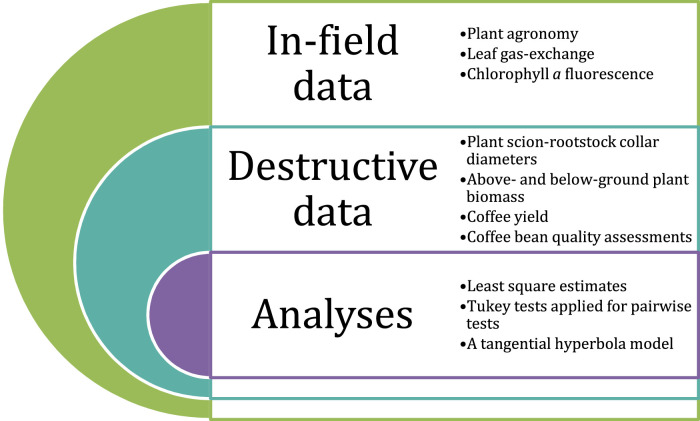
Overview of coffee data types and analyses.

Coffee plant data were measured on both living and non-living plants. These measurements generated the “in-field” and “destructive” data sources, respectively (Fig. 1). In-field data were collected several times during the experimental period. Given this, these data are accompanied by the year or specific dates of measurement. Destructive data were collected at the end of the experimental period and involved up-rooting coffee plants, separating individual plant components, and then drying and weighing each component. Analyses were conducted in R using the following packages: ‘lme4’; ‘lmerTest’; ‘pbkrtest’; ‘emmeans’; ‘ggplot2’; ‘multcomp’ (https://cran.r-project.org/web/packages/available_packages_by_name.html)

Table 1 provides a comprehensive desciption of the data sources provided. This includes the original file name, file type and a description of the data contents. Coffee types are defined as grafted- and non-grafted genotypes of *C. arabica* and a non-grafted *C. canephora* variety.

**Table 1:** Complete list of data sources.

File name	File type	Description
agronomy2018_2020.csv	CSV	Agronomic data relating to the coffee plants height, collar diameter, number, branch length, average leaf area and nodes after two and four years after plantation in the coffee field.
Cupping _La _Hilda_2021.csv	CSV	Cup quality data relating to the different coffee types assessed at the end of the trial period in 2021.
Fluorescence.csv	CSV	Chlorophyll *a* fluorescence of the coffee leaves taken on three different occasions (pre-dawn).
Graft compatibility.csv	CSV	Plant collar diameters of grafted plants (above and below the graft union sites).
Photosynthesis.csv	CSV	Light response curves and instantaneous leaf gas-exchange data of coffee leaves generated with the CIRAS-1 &3.
root2021.csv	CSV	Data of both above and belowground biomass of coffee plants (destructive measurements taken at the end of the study period).
Yield.csv	CSV	Coffee yield data
Grafting compatibility.R	R file	Statistical analyses with the “Graft compatibility.csv” data source to test whether the ratios between the scion and rootstock plant collar diameters were significantly different and therefore incompatible.
Modelling Root.R	R file	Statistical analyses with the “root2021” data source to determine whether coffee types differed based on their above and/or belowground biomasses.
Modelling quality.R	R file	Statistical analyses with the “Cupping_La_Hilda_2021.csv” data source to test whether coffee types varied in their cup quality depending on the elevation of cultivation, and tests of effects of grafting and genotype.
Modelling yield.R	R file	Statistical analyses with the “Yield.csv” data source to test whether coffee types varied in their yield based on the elevation of cultivation or graft/ genotype status.
Modelling Agronomy_2021.R	R file	Statistical analyses with the “Agronomy2018_2020.csv” data source to test whether coffee types varied in their agronomic features depending on the elevation of cultivation, and tests of effects of grafting and genotype.
Modelling FL.R	R file	Statistical analyses of the chlorophyll *a* fluorescence of coffee plants based on the “Fluorescence.csv” data source.
Modelling PN_curves.R	R file	Statistical modelling of photosynthetic light response curves based on the “Photo_combined.csv” data source.

## 4 Experimental Design, Materials and Methods

### 4.1 Field design

**Fig. 2:**
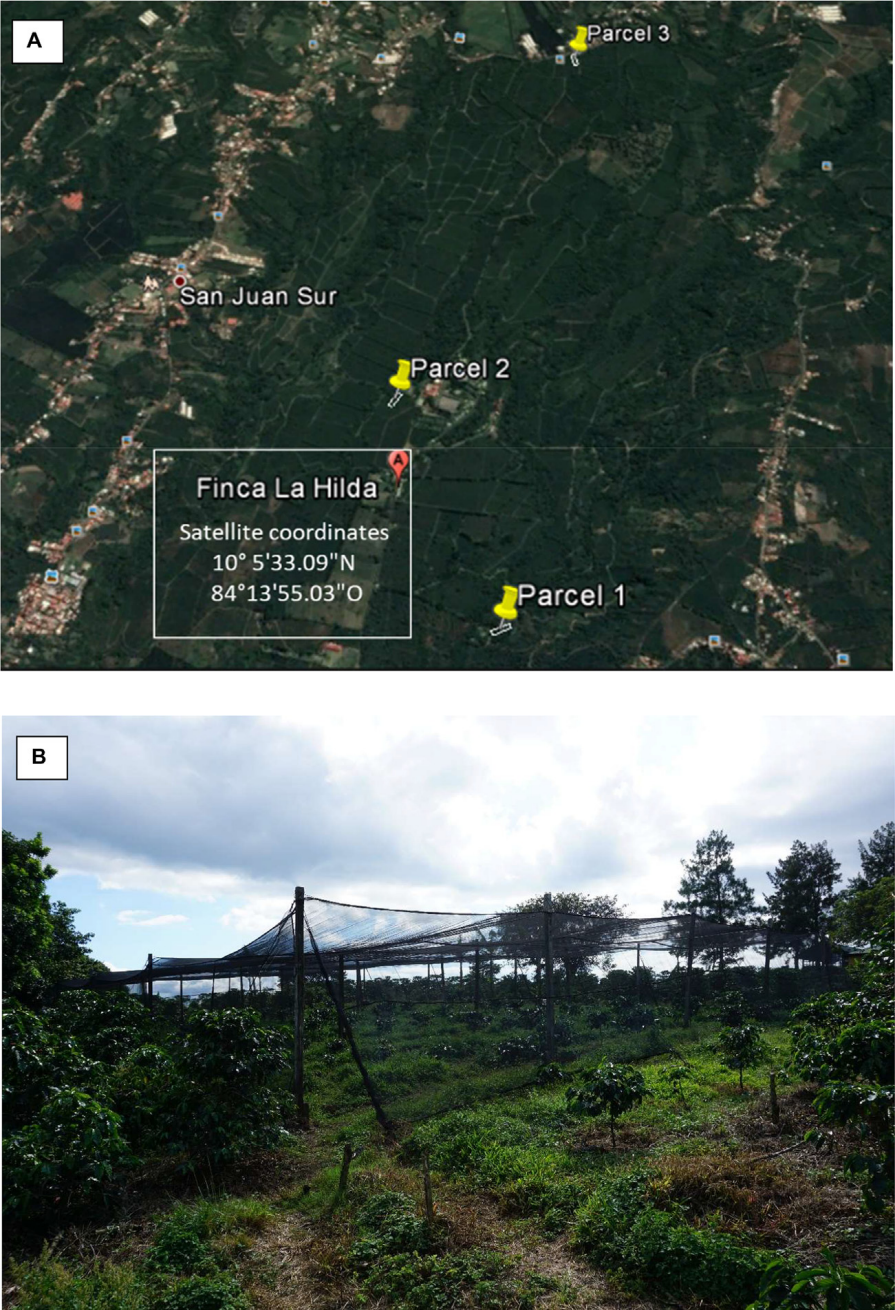
**(A)** Three field plots were used across an elevation range of 1050–1450 m.a.s.l. **(B)** Shade net was used to intercept 30 % sunlight to mimic the low-light environment of an agroforestry system.

**Fig. 3:**
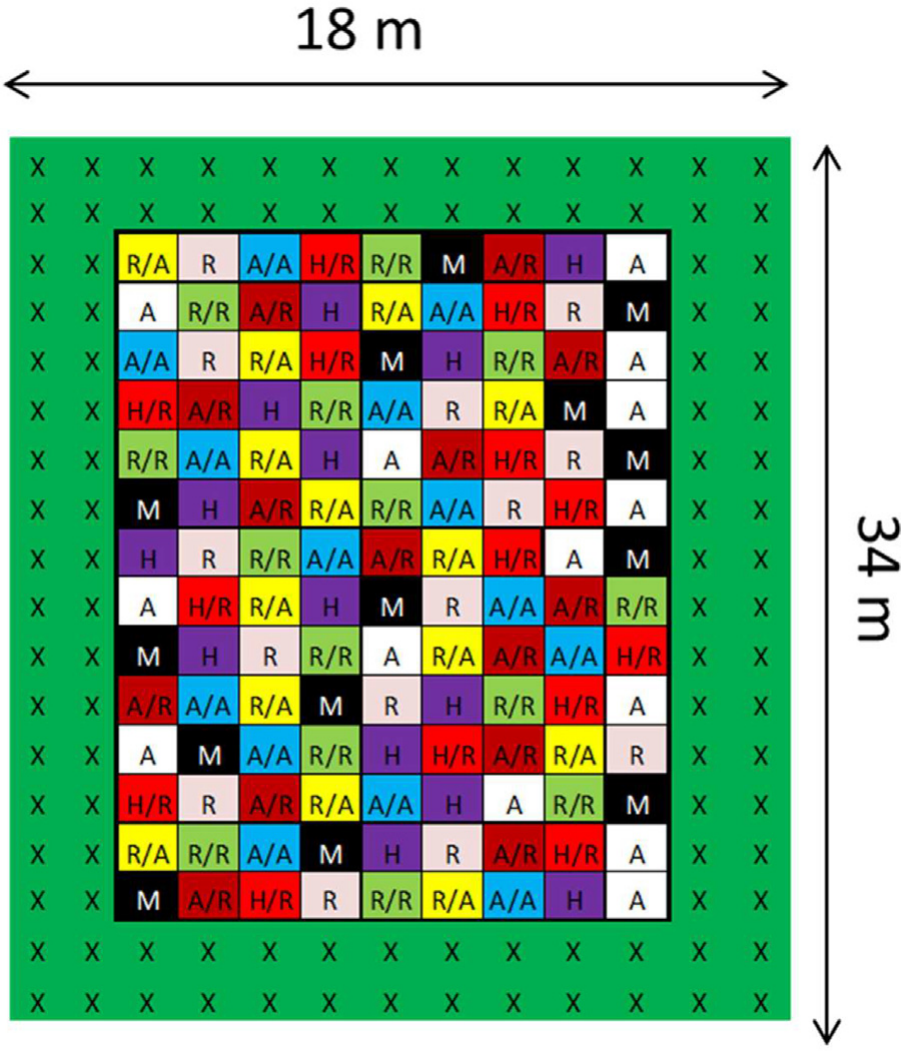
Field design of the three elevational plots belonging to the coffee grafting experiment in Costa Rica (the same design is replicated at each elevation. Letters refer to coffee type material. Colours code the coffee type: ‘Villa Sarchi’ in white (A), ‘Nemaya’ in pink (R), self-grafted ‘Villa Sarchi’ in blue (A/A), self-grafted ‘Nemaya’ in green (R/R), ‘Villa Sarchi’ grafted onto ‘Nemaya’ in dark red (A/R), ‘Nemaya’ grafted onto ‘Villa Sarchi’ in yellow (R/A), ‘H3’ in purple (H), ‘H3’ grafted onto ‘Nemaya’ in red (H/R). Coffee data represented by the black squares were omitted.

All data were collected from a commercial coffee farm (Finca “La Hilda”) in Costa Rica, San Pedro de Poas.Köppen-Geiger climate classification for the research area is Am (Tropical monsoon). Three experimental plots were designed across different elevations (1050, 1250 and 1450 m.a.s.l.). Each experimental plot was positioned in the same orientation (North–South) and the same size (18 × 34 m) (Fig. 2A). A black shade net (intercepting 30 % sunlight) was placed over coffee plants (Fig. 2B). Nine different coffee types were made by combining scions and rootstocks of various genotypes. Replicates (*n* = 14) of these coffee types were planted in a randomized manner across each plot (Fig. 3). Two rows of border coffee plants were planted around the experimental replicates. A total of 126 coffee plants were planted on each experimental plot (not including border plants). Planting densities were 1.5 × 2.0 m (*i.e.* 3000 trees ha^−1^). The daily average temperature varied between 0.5 to 1.1 °C between the elevation plots.

### 4.2 Plant material

Two coffee species were used in the experiment: *C. arabica* and *C. canephora*. Scions consisted of a *C. arabica* F1 hybrid ‘H3’ (Caturra × Ethiopian 531) and a *C. arabica* American pure line cultivar ‘Villa Sarchi’. The *C. canephora* hybrid ‘Nemaya’ was included as rootstock material. ‘Villa Sarchi’ and ‘Nemaya’ plants were produced by seed, while the ‘H3’ were derived from a somatic embryogenesis clonal propagation process (Koutouleas et al., 2023). Grafting took place one and a half months after the seed germination and three months after the somatic embryogenesis process. Different scion and rootstock combinations generated all the coffee types of the experiment *i.e.* AR, HR, RR, AA and RA (Table 2). Plants were grown in a commercial nursery under plastic tunnel for the first 6-months and then transferred to the experimental plots. In-field measurements commenced two years after plantation.

**Table 2:** List of coffee plant material.

Genetic material (incl. scion and rootstocks)	Grafting status	Treatment name (Coffee Type)
*Coffea arabica* ‘Villa Sarchi’	Non-grafted	A
*Coffea canephora* ‘Nemaya’	Non-grafted	R
F1 hybrid of *C. arabica* ‘Caturra'x Ethiopian landrace accession ‘E531′ aka. ‘H3’	Non-grafted	H
‘Villa Sarchi’ scion grafted onto ‘Nemaya’ rootstock	Grafted	AR
‘H3’ scion grafted on ‘Nemaya’ rootstock	Grafted	HR

*Note:* auto-grafted (i.e., AA and RR) and reverse-grafted (RA) combinations were used as control groups for root traits.

## 5 Methods

**Agronomic data:** manual data collection in-field or at the end of the experimental period using a measuring tape, callipers and benchtop scales.

(1) **In-field measurements**
  (a) ***Plant height:*** measured from 2 cm above the soil level up to the meristem of the orthotropic stem using a measuring tape.
  (b) ***Collar diameter of the main stem***: measured with callipers 20 cm aboveground using a 150 mm electronic digital vernier calliper (ROHS NORM 2002/95/EC, Linear, Dunstable, England).
  (c) ***Number of 2nd order plagiotropic axes branches***: those branches which grow near-horizontally from the orthotropic stem.
  (d) ***Number of total dead branches***
  (e) ***Branch length*** at ***5^th^ and 10^th^ order plagiotropic axes***
  (f) ***Leaf area*** at branch level 5 and 10 (method according to Bryant and Kothmann [4]).
  (g) ***Number of fruiting nodes*** visually determined per plant [5].
  (h) **Leaf gas-exchange**: Simultaneous measurements of leaf CO_2_ assimilation and transpiration reflect changes in both stomatal conductance and mesophyll capacity for photosynthesis [6]. Measurements of leaf gas exchange including transpiration (*E*), net photosynthesis (*P*_net_) and stomatal conductance (*Gs*) were performed using a portable photosynthesis system (CIRAS-1 and 3 (PP-systems, Hitchin, UK). leaf gas-exchange was conducted on the fully open, third plagiotropic leafsurface. The PLC3 and PLC-B Universal Leaf Cuvette was used for all measurements. The cuvette flow rate was set to 300cc min^−1^, leaf area was 4.5 and 2.5 cm^2^ (respectively). Photosynthetic active radiation (PAR) was based on the natural light present in the field; CO_2_ range was between 400–420 ppm; H_2_O % was set to 80 %; leaf temperature was set to air temperature. There was a strong diurnal effect found in the field therefore the measurements were performed between 07:00–1:00 pm at the end of the rainy season (October–December) on clear sunny days. The number of plants sampled per coffee type and per elevation was six. Single point measurements (SPMs) were recorded at ambient light when the internal cuvette environment was stable (usually within 1–2 minutes). A total of 1171 instantaneous point measurements of leaf gas-exchange were performed on the coffee types across the three elevations.
    Light Response Curves (LRCs) were developed and statistical tests were based on residual values from the light response curves above and below PAR 300. This was conducted because the light saturation point of *C. arabica* tends to plateau between 300–600 PAR with the lower range being specific to shaded coffee plants (Kumar & Tieszen, 1980). LRC parameters (Table 3) were derived based on three regions of the constructed LRCs and subjected to statistical analysis as well.

**Table 3:** Leaf gas-exchange parameters.

Parameter	Description
*P*_net_	Daily net photosynthesis (μmol of CO_2_ fixation per m^−2^ s^−1^). Measured by instantaneous point measurements.
*Vc*_max_	Maximum carboxylation capacity of enzyme ribulose-1,5-bisphosphate carboxylase / oxygenase (RubisCO) (μmol CO_2_ m^−2^ s^−1^) signified by the plateau region of LRCs.
ΦCO_2max_	Maximum quantum yield of photosynthesis (mol CO_2_ photon^−1^) derived from the linear region of the LRCs.
  (i) **Chlorophyll fluorescence:**
    Chlorophyll fluorescence analysis provides an indication of the fate of light energy intercepted by the plant leaf. Light energy can either be used for photosynthesis (photochemistry), dissipated as heat, or re‐emitted as light in the form of chlorophyll fluorescence [7]. By determining the yield of chlorophyll fluorescence, efficiency of photochemistry and heat dissipation on the leaf level can be estimated [7].
    The Chlorophyll *a* fluorescence parameters (Table 4) were recorded with a Pocket-PEA handheld fluorometer (Hansatech Instruments Ltd., Norfolk, England) settings at a saturating, white light pulse of 3500 µmol m^−2^ s^−1^ and 3 s duration. Measurements were performed on the middle of the fully open, third plagiotropic leaf pairs, eight hours after sunset (*ca.* 00:00–03:00am). A total of 4 measurements (replications) were recorded for each genotype on three separate occasions. A total of 318 measurements were collected for the coffee types across the three elevations.

**Table 4:** Chlorophyll *a* fluorescence parameters.

Parameter	Description
PI*abs*:	Performance index for energy conservation from photons absorbed by photosystem (PS) II antenna, to the reduction of plastoquinone b (Q*_b_*)
Fv/Fm:	Photosynthetic performance (maximum quantum yield = φp0 = TR/ABS) and used as a simple proxy to estimate photosynthetic oxidative stress damage (Kasajima, 2017).
ABS/RC:	Absorption per reaction centre (RC) in antenna pigment chlorophyll
TR*0*/RC:	Calculated trapping at time zero per RC (energy flux transmitted per RC)
DI*0*/RC:	Heat dissipation at time zero per RC (energy flux dissipated per RC)
ET/CS*0*:	Electron transport at time zero per cross section (CS) (plastoquinone Q*_a_* to Q*_b_*)
PhiE*0*:	Electron transport (Q*_b_* to PS I cyclic electron flux)

**Fig. 4:**
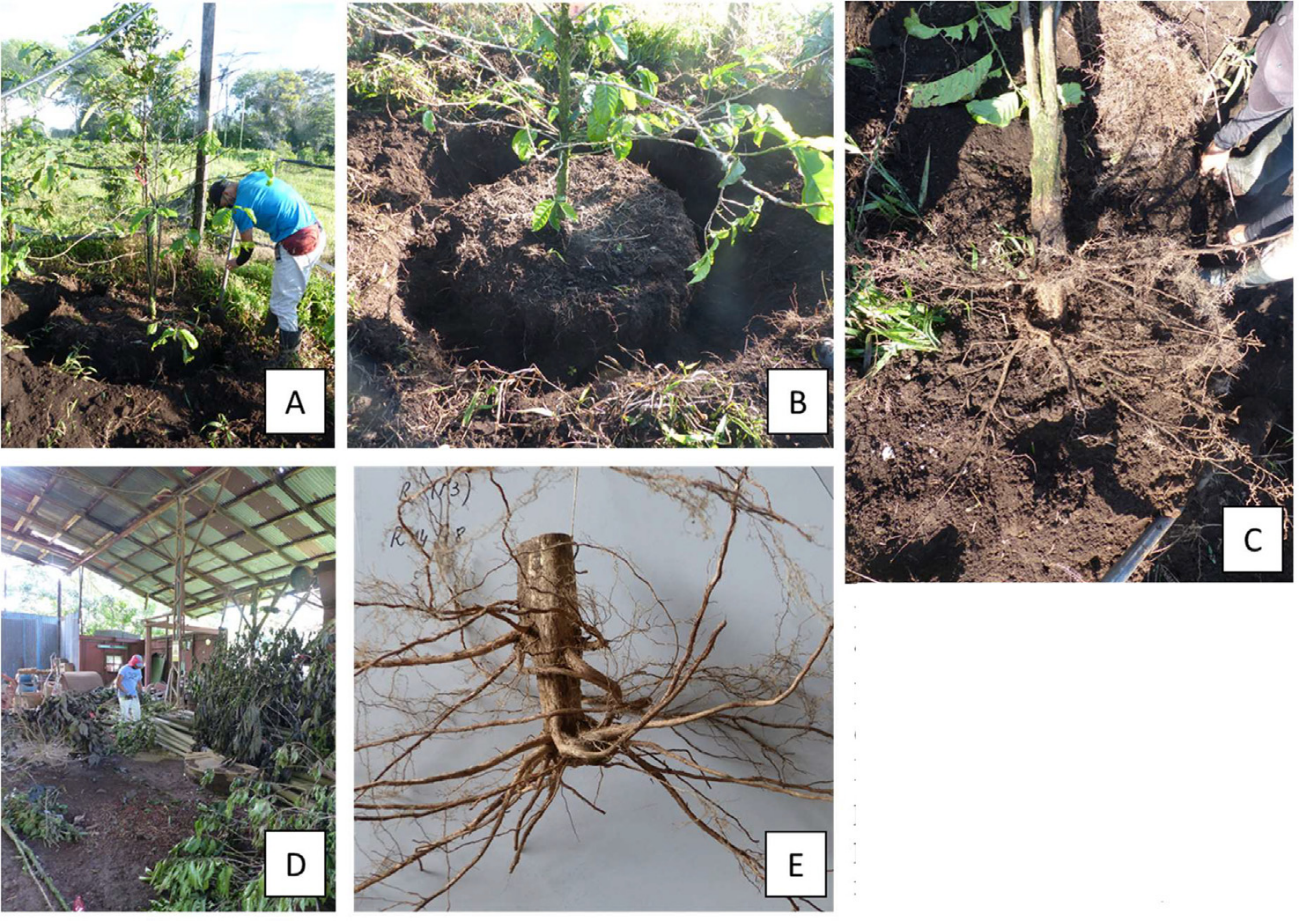
(A) excavation of coffee plants. (B) 80 cm whole around base of the plants. (C) root systems after excavation. (D) drying process of the plants in a warehouse. (E) dry tap root and secondary roots.
  (c) **Grafting compatibility:** measurements of the thickness of the diameter of the graft union versus the scion plant collar was conducted on several grafted plants through the determination of a ratio (Fig. 5). The ratio was used to detect compatibility of the scion with the rootstock. The larger the ratio, the less compatible the graft combination.

(2) **Destructive measurements**
  (a) **Coffee yield:** Coffee berry yield per coffee type, number of harvests, weight of green beans per harvest and dates of harvest. Beans were processed using the wet method according to Joët et al. [8].
  (b) **Above-belowground biomass:** In order to study the root system and the ratio between the dry weight of the root system and that of the aerial system of grafted and non-grafted plants, seven plants per coffee type were excavated from each plot under the shade condition (84 plants in total). Coffee plants were excavated to a depth of approximately 80 cm and a diameter of 80 cm around the plant base (Fig. 4). The complete plant were air dried on a wire in a warehouse for three weeks. The root systems were washed with water to remove any residual soil. The aerial part of the plant (*i.e.* the main stem, the secondary branches and the leaves), and the root system (separated in tap root and the secondary roots) were weighed individually to determine above and below ground biomass data.

**Fig. 5:**
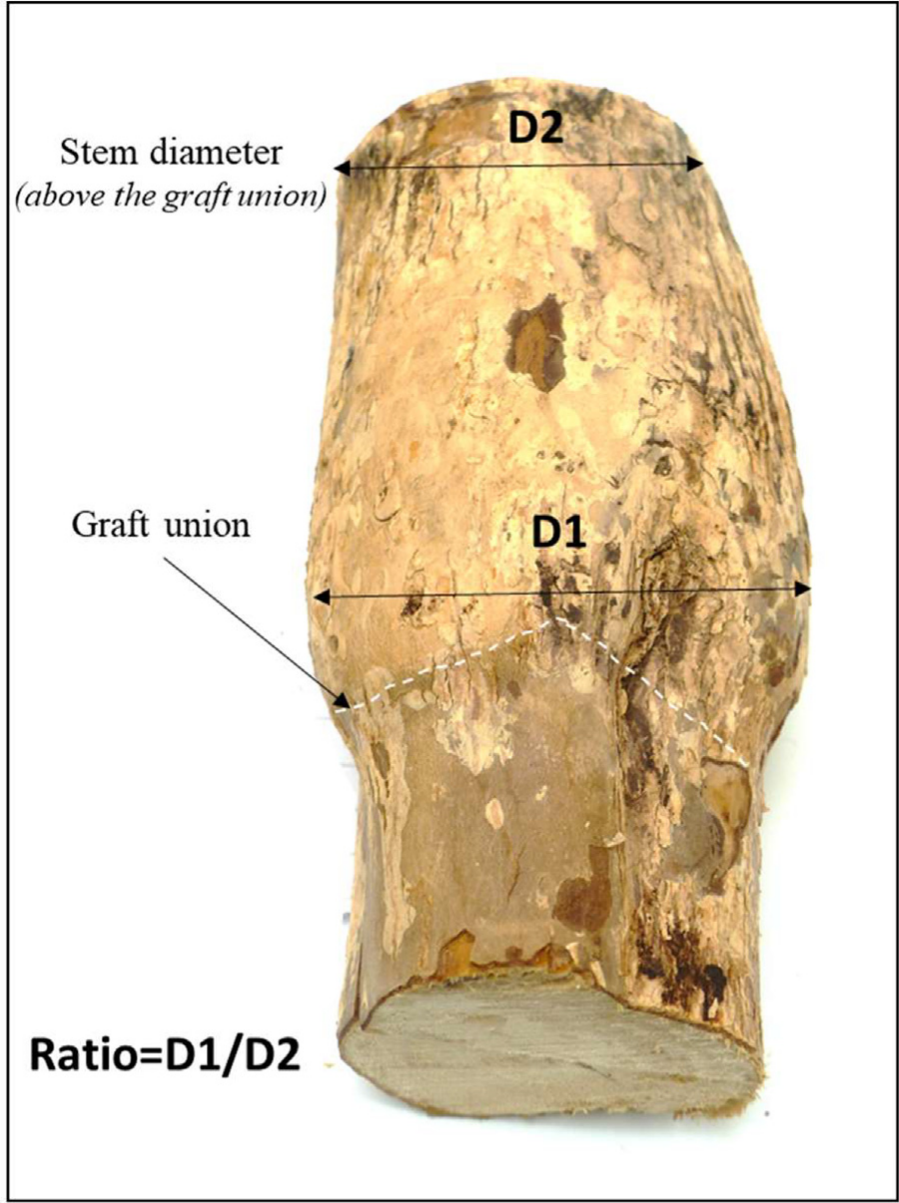
Ratio determination on grafted plants taken into account the diameter (D) of the graft union and the scion plant collars. The scion collar diameter (D1) was measured at the broadest place above the graft union.
  (d) **Bean traits:** coffee beans were sieved with mesh sizes 15/64 to 17/64 inches and physically assessed for the presence of peaberries and other defects. The resulting data were defect %; Sieve number 17, 16 and <15; WB100; Peaberry %, Water % and SCA note.
  (e) **Cup quality:** coffee cup testing followed the standard Specialty Coffee Association (SCA) protocol [9] and was conducted by two expert testers from ECOM company (Cafinter SA in Costa Rica) (Fig. 6).

**Fig. 6:**
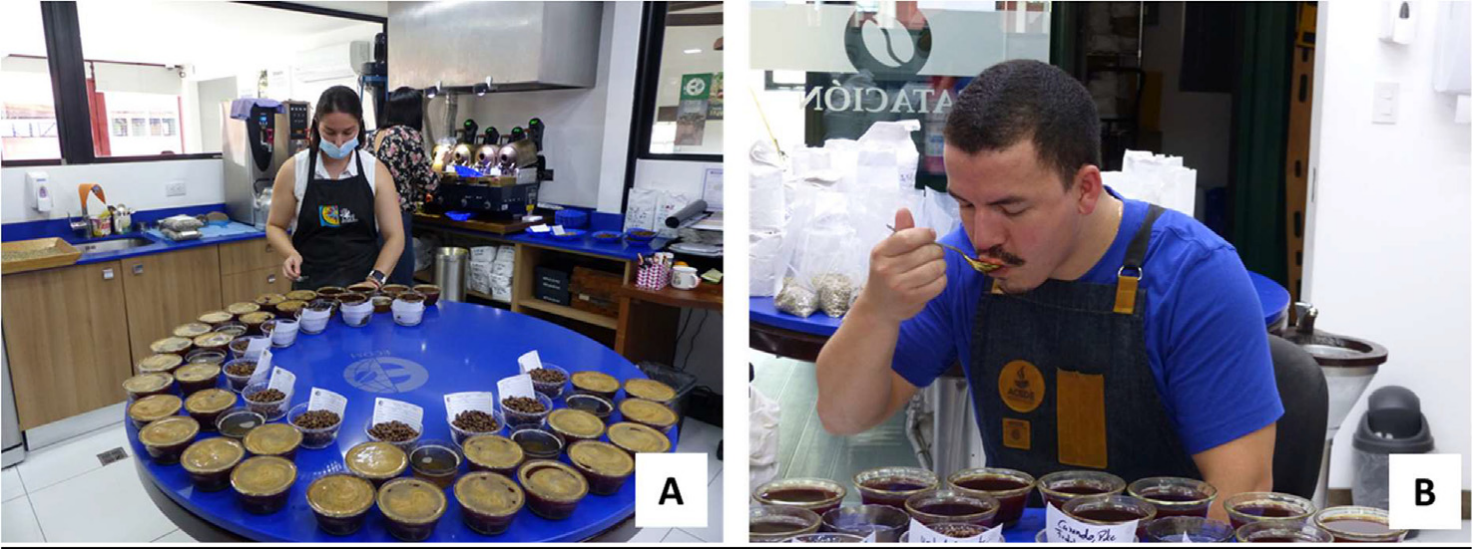
Cupping Session at Cafinter S.A. company (ECOM group). **(A)** Preparation of the samples **(B)** Cupping session by Mr Rudy Azofeita (Q Grader SCA).

## Ethics Statements

N/A.

## CRediT authorship contribution statement

**Athina Koutouleas:** Data curation, Data curation, Methodology, Formal analysis, Writing – review & editing. **Conor Blunt:** Data curation, Writing – review & editing. **Aljoša Bregar:** Data curation, Writing – review & editing. **Jon Kehlet Hansen:** Data curation, Methodology, Formal analysis, Writing – review & editing. **Anders Ræbild:** Data curation, Methodology, Formal analysis, Supervision, Writing – review & editing. **Hervé Etienne:** Writing – review & editing. **Frédéric Georget:** Conceptualization, Data curation, Data curation, Methodology, Formal analysis, Writing – review & editing.

## Data Availability

Plant physiology, yield and quality data of interspecific grafted Coffea arabica across an elevation gradient (Original data) (Open Science Framework).
